# Exploration of covalent-organic frameworks and metal-organic frameworks for drug delivery applications

**DOI:** 10.3389/fchem.2026.1798045

**Published:** 2026-03-27

**Authors:** Yuhao Sheng, Kunfang Ma

**Affiliations:** 1 School of Pharmacy, Nanjing Medical University, Nanjing, China; 2 Pharmaceutical Experiment Teaching Center, School of Pharmacy, Nanjing Medical University, Nanjing, China

**Keywords:** cancer therapy, covalent-organic frameworks, delivery system, drug carrier, metal-organic frameworks, synthesis approach

## Abstract

Covalent-organic frameworks (COFs) and metal-organic frameworks (MOFs) have been a promising carrier for drug delivery because of excellent biocompatibility, developed porous structure and sufficient interactions of host-guest molecules. This paper mainly reviews latest research progress of COFs and MOFs including their synthesis approaches and applications in drug delivery. Specifically, COFs as drug carriers in chemotherapy, photodynamic therapy, photothermal therapy, synergistic therapy, anti-bacteria, and anti-inflammatory were systematically presented. MOFs in pharmaceutical drug delivery, controlled-release drug delivery, stimulus response and targeted drug delivery systems are introduced. The advantages and disadvantages of COFs and MOFs in synthesis and drug delivery performances was systematically compared. The future development trends of COFs and MOFs for drug delivery applications are discussed.

## 1 Introduction

Drug delivery system is to load drugs by using carriers to deliver appropriate amounts of drugs to organisms at appropriate times. The release and distribution of drugs can be effectively controlled at specific locations in organisms. The delivery of drugs to the target can be enhanced by drug delivery system to minimize the loss of drugs in the delivery process. Drug delivery system presents multiple advantages including improved drug stability, decreased drug degradation, reduced adverse drug reactions, controlled and targeted drug release (Hosny et al., 2025). The compliance of patients can be promoted accordingly to improve the health of patients. Since the prevalence of nanotechnology in the field of medicine, it has almost completely focused on tumor-targeted drug delivery.

Drug delivery technologies such as oral preparation, transdermal preparation and injection have been developed successively by 1952 (Valenzuela et al., 2021). However, the carrier system itself may be potentially harmful and bring risks to patients. An effective drug delivery system needs high drug loading, sustained drug release, local control of target release and solubility in water. Moreover, drug delivery system must be non-toxic and preferably have good biocompatibility.

Covalent-organic frameworks (COFs) and metal-organic frameworks (MOFs) are two crucial porous materials with characteristics of sufficient active sites, high surface area, and regular porous structure (Scicluna and Vella-Zarb, 2020). Specifically, COFs are crystalline porous materials composed of organic molecules connected by covalent bonds, which has a high degree of order and predictable pore structure. COFs are mainly composed of light elements with good chemical stability and thermal stability (Feng et al., 2020). MOFs are constructed by formed by coordinating organic ligands with metals (Filiz et al., 2025). MOFs have a high structural diversity, and can form a variety of different frame structures through different combinations of metal and organic ligands (Han et al., 2025). However, there are still some critical challenges currently faced in the field.

As recent research hotspots in the field of biomedicine, the biocompatibility of MOFs and COFs is crucial to determine their application potentials. For MOFs, potential toxicity mainly comes from the metal nodes in their frameworks. Therefore, essential metals for the human body or metals with lower toxicity (e.g., Fe, Zn, Cu) should be preferentially selected to construct MOFs (Yan and Lin, 2025). Meanwhile, MOFs with endogenous and biodegradable organic ligands can ensure the decomposition of MOFs into non-toxic products, which can be metabolized or excreted by the human body after completing its task (Abbas et al., 2025). It is necessary to precisely control these activities so that they can exert therapeutic effects without causing damage to normal tissues. COFs have unique advantages in terms of biocompatibility due to their organic structure entirely connected by covalent bonds. No metal is existed in the frameworks of COFs, which fundamentally eliminates people’s concerns about the long-term retention or leakage of heavy metal ions, enabling an inherently excellent biocompatibility (Kang et al., 2025; Zhang et al., 2025a).

*In vivo* stability of MOFs and COFs is highly concerned for the application in drug delivery. Metal-ligand coordinating bond strength decides *in vivo* stability of MOFs. Under physiological conditions (e.g., PBS buffer solution and culture medium), the crystal structure of some MOFs can be maintained for a period of time. However, MOFs face challenges in certain complex biological environments due to the reversible coordination bonds (Khan et al., 2026; Sang et al., 2026; Yu et al., 2026). MOF particles tend to aggregate in body fluids and may be quickly cleared by the immune system. This is one of the main obstacles for their clinical application. By using surface modification (e.g., coating with BSA protein), the colloidal stability can be significantly enhanced, thereby prolonging circulating duration. Coordination bonds of MOFs enable their responsive degradation under specific conditions (e.g., acidic pH, highly concentrated glutathione or the presence of phosphate) (Patil et al., 2025; Yu et al., 2025). This allows for their applications in precise release of drugs at tumor and other lesion sites. The degraded products are non-toxic and capable of being metabolically excreted. COFs are connected by strong covalent bonds including imine bonds and borate ester bonds, which endows them with excellent chemical and thermal stability. Their structural integrity can be maintained in the physiological environment, effectively protecting the drugs and preventing their leakage before reaching the lesion site. However, high stability of COFs in body’s circulation may also lead to slow degradation in the body and difficulty in clearance, posing a risk of long-term retention (Kumar et al., 2025; Zhang et al., 2025b). How to balance the stability and degradability of COFs is currently a major challenge in the field of drug carriers by COFs.

Moreover, the scalability of COF/MOF synthesis is another crucial issue. For scalable MOF synthesis, the traditional solvothermal method relies on severe synthetic conditions. When scaled up, heat and mass transfer efficiency drops sharply, resulting in inconsistent product quality and poor batch reproducibility (Li et al., 2026; Yang et al., 2025). Although there are new methods including mechanical chemistry and flow chemistry, how to achieve high yield, high crystallinity and green non-pollution simultaneously in large-scale production of MOFs remains a major challenge. For long-term hindered large-scale producing COFs, commonly used solvothermal method can produce high-quality COF crystals. This method suffers from high temperature, high pressure, long reaction time (often requiring several days), and a large amount of toxic organic solvents (Liang et al., 2025; Zhang et al., 2025c). The single production yield of this method is usually only in the range of milligrams to grams, which cannot meet the efficiency and output requirements for industrial applications. The larger the reactor, the worse the internal heat and mass transfer efficiency becomes, resulting in uneven reactions and significant differences in product quality among batches.

**FIGURE 1:**
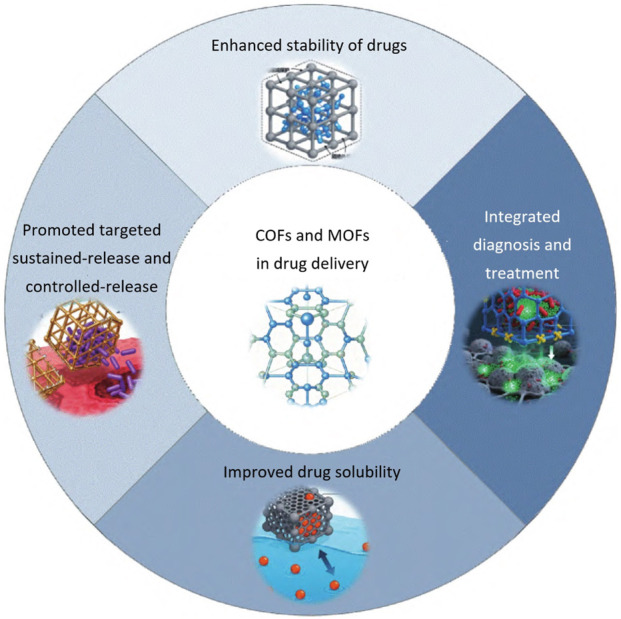
Application advantages of COFs and MOFs in drug delivery systems.

This paper presented systematical review synthesis and drug delivery application of COFs and MOFs as illustrated in Figure 1. The preparation approaches of COFs and MOFs were briefly introduced first. Then the drug delivery using COFs and MOFs were presented via providing latest studies and systematical comparison of advantages/disadvantages.

## 2 Synthesis methods of COFs and MOFs

### 2.1 Synthesis methods of COFs

#### 2.1.1 Solvothermal synthesis

In solvothermal reaction, general synthesis steps were as followings. Monomer and solvent were added into Pyrex tube. The mixture was frozen with liquid nitrogen, vacuumed, and thawed. By repeating the above operations for three times, oxygen was removed. As the reaction carried out in sealed pyrex tube for given times, large numbers of insoluble substances were produced. The COF materials could be obtained through a series of purification.

**FIGURE 2:**
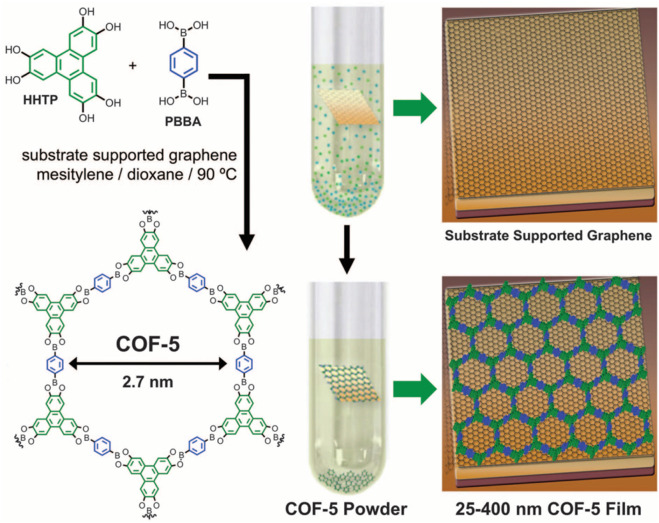
Solvothermal synthesis of COF-15 (Colson et al., 2011). PBBA and HHTP were condensed on graphene surface. Precipitation of COF-5 powder was performed to prepare COF-5 film. Reproduced from (Colson et al., 2011). Copyright 2011 Science.

Common reaction temperature for COF synthesis is 85 °C-120 ^o^C (Côté et al., 2007; El-Kaderi et al., 2007; Fang et al., 2014). Reaction time can be changed from 1 day to 9 days. To get better yield and performance, most COF materials need to be reacted for at least 3 days. Solvents with various species and ratios are selected in different reaction systems. Both solvent species and ratio exert an influence on the morphology, porosity and property of the prepared COF products. Solvothermal method can not only synthesize COF powders but also thin COF films. In 2011, Dichtel et al. synthesized two-dimensional COFs on monolayer graphene via solvothermal method (Figure 2) (Colson et al., 2011). This method comprised the following steps: a substrate covered with single-layer graphene was putted into a reaction system for synthesizing COF-5, and reacted at 90 °C for a certain time to obtain a highly oriented COF-5 on single-layered graphene. This method can also be used to synthesize other highly oriented two-dimensional COF materials, such as NiPc-PBBA COF.

#### 2.1.2 Ionic thermal synthesis

Only triazine-based COFs were prepared using this method. In 2008, Thomas and Kuhn synthesized CTF-1 with following procedures (Kuhn et al., 2008). Monomer 1,4-dicyanobenzene and metal salt zinc chloride were added into an ampoule bottle. The ampoule bottle was vacuumed, sealed and reacted at 400 °C for 40 h. When the temperature of the system was cooled to room temperature, the ampoule bottle was opened, and the obtained solid was subjected to a series of treatments to obtain CTF-1. In the reaction process, molten zinc chloride catalyzed the reversible cyantrimerization reaction. In 2010, Thomas and Kuhn used the same strategy to synthesize another triazine COF material (i.e., CTF-2) from 2,6-dicyanonaphthalene in molten zinc chloride (Bojdys et al., 2010). However, the harsh reaction conditions of this method and high requirement of thermal stability of synthetic building unit due to high reaction temperature restrict the wide application of this synthesis approach.

#### 2.1.3 Microwave-assisted synthesis

Microwave-assisted method (i.e., solvothermal synthesis via microwave heating) heated object without the process of uniform heat conduction in external circumstances (Kappe, 2004; Kappe and Dallinger, 2009). Moreover, polar solvents to achieve better heating effect is needed. Cooper et al. prepared two-dimensional and three-dimensional COFs (2019 cm^2^/g) via this approach for the first time, which was 200 times faster compared with the duration via traditional solvothermal method (1,590 cm^2^/g) (Campbell et al., 2009). In 2015, TpPa-COF was successfully synthesized in a microwave reactor at 100 °C for 60 min (Wei et al., 2015). The stability and crystallinity of TpPa-COF were both high, presenting a good potential in the industrial fabrication.

#### 2.1.4 Mechanical grinding synthesis

**FIGURE 3:**
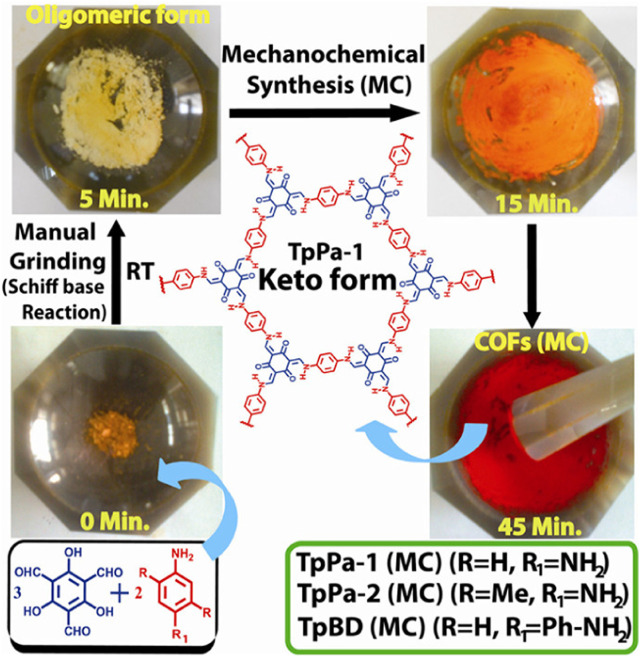
Illustration for mechanochemically preparing TpBD (MC), TpPa-1 (MC) and TpPa-2 (MC) (Biswal et al., 2013). Schiff base reaction was induced by manual grinding. Benzidine, dimethyl-p-phenylenediamine, and phenylenediamine were milled with triformylphloroglucinol to obtain a dark-red product. The synthesis of COF could be completed totally with manual grinding for 40 min. Reproduced from (Biswal et al., 2013). Copyright 2013 American Chemical Society.

Banerjee et al. first reported mechanical grinding synthesis of highly stable two-dimensional TpPa-1 (MC), TpPa-2 (MC), and TpBD (MC) without solvents (Figure 3) (Biswal et al., 2013). The BET specific surface areas of these three COFs reached 61, 56 and 35 m^2^/g, respectively. While those of these three COFs prepared via the traditional solvothermal method were 535, 339 and 537 m^2^/g, respectively. Mechanical grinding method for synthesizing COFs presented advantages including facile rpecedure, rapidity, environmental friendliness and large-scale production. Few instances for synthesizing COFs using this method are reported so far.

#### 2.1.5 Heating reflux synthesis

In 2006, Lavige group added 1,3, 5-triphenylboronic acid and 1,2,4, 5-tetrahydroxybenzene to a mixed solvent containing methanol (2%) and tetrahydrofuran, and then heated and refluxed for 72 h in a nitrogen environment to synthesize the two-dimensional COF-18A (Tilford et al., 2006). This is the first case of synthesizing COFs using the heating reflux method. Subsequently, this group used the same synthetic method to synthesize COF-11A, COF-14A and COF-16A respectively using 1,3,5-triphenylboronic acid and various 1,2,4,5-tetrahydroxybenzene derivatives as raw materials, thus pioneering the synthesis of COFs by heating reflux method (Tilford et al., 2008).

In 2008, Jiang team added pyrene-2,7-diboronic acid (PDBA) and hexahydroxytriphenylene (HHTP) to substances containing 1, 4-dioxane and isotriphenylene (volume ratio 1:1). A two-dimensional COF with blue fluorescence (TP-COF) was successfully synthesized by heating and stirring at 85 °C for 3 days with an argon atmosphere (Wan et al., 2008). TP-COF presented a regular banded structure with a length at the micrometer level.

#### 2.1.6 Room-temperature solution synthesis

In 2015, Yan and Yang et al. successfully synthesized the COF material TpBD in a solution at room temperature (Yang et al., 2015). The synthesis steps were as follows: by stirring benzidine (BD) and triformylphloroglucinol (Tp) with ethanol solvent, TpBD was obtained by stirring at room temperature for 30 min. Specific surface area of TpBD synthesized by this method reached 885 m^2^/g, being higher than that of TpBD synthesized by solvothermal method (537 m^2^/g). It was also higher than the specific surface area of TpBD synthesized by mechanical grinding (35 m^2^/g). COFs preparation by the room-temperature solution method exhibits high thermal stability and acid-base stability with a poor universality. Therefore, up to now, there are still few successful examples of synthesizing COFs with superior properties by room-temperature solution approach.

### 2.2 Synthesis methods of MOFs

#### 2.2.1 Hydrothermal or solvothermal synthesis

“Hydrothermal” refers to a chemical reaction that occurs in the presence of water (a dipole proton solvent) at a temperature higher than the boiling point and under a pressure greater than 1 atmosphere. “Solvothermal” indicates that a chemical reaction occurs in the presence of an organic solvent (protonic or aprotic solvent). Hydrothermal or solvothermal methods can flexibly control the size and magnitude of MOFs by regulating the stoichiometric ratio, concentration of reactants or adding regulators, etc. Qian et al. controlled the particle size and morphology of ZIF-67 by the molar ratio of Co source, 2-methylimidazole to Co^2+^ and the type of solvent (Qian et al., 2012). With the decrease of reactant concentration, product particle size rose gradually, and MOFs morphology tended to be nearly spherical in shape.

#### 2.2.2 Ultrasonic and microwave-assisted synthesis

Microbubbles produced by ultrasonic waves would expand and close rapidly. At the moment the bubbles close, a powerful shock wave is produced, creating localized high pressure and temperature surround bubbles. Ultrasonic-assisted synthesis usually refers to use the energy of ultrasonic waves to provide the energy required for reaction. Li et al. efficiently prepared Ni-based MOF [Ni_2_(OH)_2_(C_8_H_4_O_4_)] with a layered microporous structure within 2 h using an ultrasonic-assisted solvent method (Li et al., 2017).

The microwave-assisted synthesis method uses microwaves to provide energy to the reaction system, which can promote the rapid dissolution of metals and organic ligands with poor solubility, thereby shortening the nucleation period. Bashar et al. synthesized a novel Fe_3_O_4_/Zn MOF magnetic nanomaterial within 20 min by microwave-assisted method, which can be used as a high-performance antibacterial agent and magnetic nanocatalyst (Bashar et al., 2022). Chen et al. applied this fast scheme to synthesize functional MOF-74(Ni) products within 60 min (Chen et al., 2019).

#### 2.2.3 Mechanical chemical synthesis

In mechanical grinding, solid reactants are converted into products through grinding, sieving or shearing without the need for excessive organic solvents (Martinez et al., 2023). Environmental friendly mechanical grinding preparation with short interaction time was utilized to prepare several classic MOFs, such as zeolite azo organic frameworks (ZIFs), iso-network MOFs (IRMOFs) (Prochowicz et al., 2015), HKUST-1 (Stolar et al., 2017), and derivatives of UiO-66 and UiO-67 (Ali-Moussa et al., 2017; Fidelli et al., 2018). Recently, Jin et al. have integrated mechanical grinding methods and MOFs defect engineering theories, enabling the rapid and green synthesis of gram-scale chiral MOF material Cu-BTC-M within 2 h (Jin et al., 2022). The product features stratified micro/mesoporous pores and excellent asymmetric catalytic activity.

For hot melt extrusion method, extrusion is a continuous process. During the extrusion process, the material is forced to pass through a confined space and is simultaneously subjected to intense shear mixing effects. In recent years, hot melt extrusion has been found to be effective for solvent-free mechanochemical synthesis of MOFs and for industrial mass production. Kriesten et al. prepared flexible MOF-53 and MOF-53-NH_2_ by hot melt extrusion using methylcellulose as the binder, which could completely retain their respiratory behavior (Kriesten et al., 2019).

#### 2.2.4 Microemulsion synthesis

Surfactant assisted two immiscible liquids would form a stable dispersion thermodynamically as microemulsions. They were reactors for preparing nanostructures, which could confine reactions in nanoscale and adjust MOF size controllably. Recently, this method has been used to controllably grow and synthesize core-shell nanostructures on the surface of nanorods (e.g., Au@MIL-88A) using microemulsion-COOH-terminated Au nanorods as the core to control the growth of surface crystal MOF. Microemulsion synthesis method is simple and highly repeatable, and has prepared MOF structures with high dispersion and uniformity (i.e., an average diameter of 89 ± 3 nm and star-shaped morphology) (Shang et al., 2017).

#### 2.2.5 Electrochemical synthesis

Electrochemical synthesis of MOFs included cathodic deposition and anodic dissolution. Electrochemical synthesis continuously provides metal ions required for the formation of MOFs with controlled addition of organic ligands and electrolytes (Li et al., 2022). Compared with the traditional high-temperature synthesis method for preparing MOF-5, the cathodic electrodeposition method could obtain MOF-5 crystals within 15 min at room temperature (Varsha and Nageswaran, 2020). However, due to the limitations of large-scale electrochemical equipment and large-area metal electrodes, electrochemical methods are generally not suitable for industrial production.

### 2.3 Advantage/disadvantages comparison of synthetic methods

Advantage and disadvantages of synthetic methods for COFs and MOFs were compared as Tables 1,2 shown, respectively.

**TABLE 1:** Comparison of COF synthesis characteristics.

Representative COFs	Methods	Reaction conditions	Product yield	Porosity	Scalability	Advantages	Disadvantages
COF-102 COF-103 COF-105	Solvothermal method	DMF/dioxane/trimethylbenzene, >120 °C, >72 h	—	>1,000 m^2^/g	Medium	Simple Controlled process	Large amounts of organic solvents used Large consumed energy due to high temperature/pressure
CTF-1 CTF-2	Ionic thermal method	ZnCl_2_, 400–700 °C; 20–116 h	—	2,404 m^2^/g	Low	Short reaction time	High amounts of residual ions High reaction temperature
COF-5 COF-102 TpPa-COF	Microwave-assisted method	1,4-Dioxane/Bis-terphenyl, <1 h	—	2,600 m (Valenzuela et al., 2021)/g	High	Fast reaction speed Facile operation	Special equipment required Dangerous energy accumulation in short periods
TpPa-1 TpPa-2 TpBD	Mechanical grinding method	170 °C, 20 min	98%	960 m^2^/g	Medium	Two synthetic steps Extremely short time High yield	Low product crystallinity Small scope of application
COF-11 COF-14 COF-300	Heating reflux method	9–12, 1,4-Dioxane	>90%, >28 kg/m3/day	400 cm^3^/g	High	High product crystallinity	Long reaction time Large amounts of solvents
TpBD	Room temperature solution method	Room temperature, 30 min, ethanol	—	885 m^2^/g	Low	Facile operation No heating High safety Low pollution	Long reaction time Low precise product control Wide particle size distribution Poor repeatability

**TABLE 2:** Comparison of MOF synthesis characteristics.

Representative MOFs	Methods	Reaction conditions	Product yield	Porosity	Scalability	Advantage	Disadvantage
ZIF-8 Mn-ZrMOF KAUST-7/8 MIL-101(Cr)	Hydrothermal and solvothermal method	80 °C–250 °C, 2–72 h, DMF	83.3%	>1,000 m^2^/g	Medium	Simple and controllable process	Large amounts of organic solvents used Energy consumption due to high temperature and pressure conditions
UIO-66 Mn-UMOFNs MOF [Ni_2_(OH) _2_(C_8_H_4_O_4_)]	Microwave and ultrasonic assisted method	100–120 °C, 3–120 min, DMF	2,241 kg/m^3^/day	1,661 m^2^/g	High	Quick reaction speed High reaction efficiency Controllable product size	Special synthesis equipment required Dangerous energy accumulation in short periods
UIO-66 5Fu@MIL-100 MOF-505 HKUST-1	Mechanical grinding method	45–75 min, few methanol	High	35–61 m^2^/g	Medium	Not a lot of solvents required Quick reaction speed and high yield Environmental protection	Low product crystallinity Small scope of application
UiO-66-NH_2_ MOF-74 Fe-MIL-88A Cu-MOF	Microemulsion method	DMF, water, room temperature	—	30–50 nm, 10–20 nm	Low	Shortened cycle of synthesis Controlled crystals’ shape and size	Complicated operation Difficult to industrialize
ZIF-8 ZIF-71 ZIF-67 Co-MOF	Electrochemical method	Mild, room temperature	—	High	Low	Mild reaction conditions Easy operation Short reaction time	Side reactions Metal co-deposition in cathodic electrosynthesis Difficult to industrialize

## 3 Applications of COFs and MOFs in drug delivery

### 3.1 Applications of COFs in drug delivery

**FIGURE 4:**
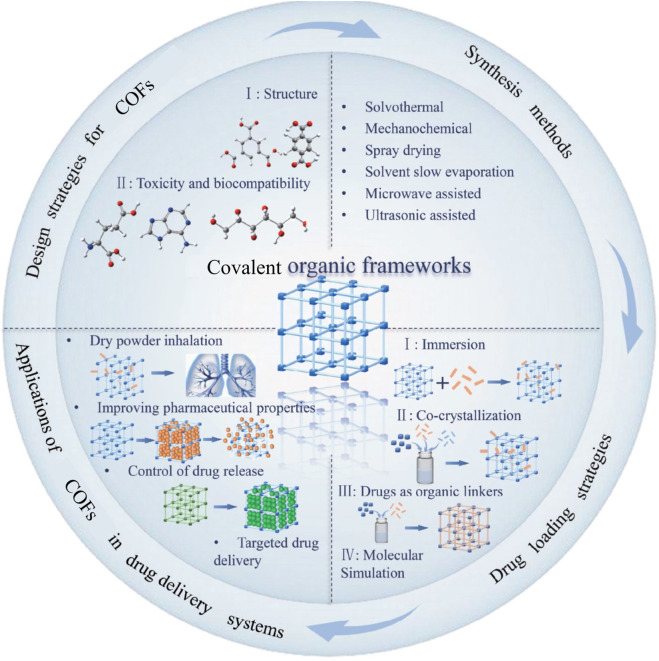
Applications of COFs in drug delivery systems.

The application of COFs in drug delivery is illustrated in Figure 4. The broad utilization of COFs in drug delivery including anti-tcancer drugs, anti-bacteria drugs, and anti-inflammatory drugs was presented in following content.

#### 3.1.1 COFs in the delivery of anti-tumor drugs

In recent years, traditional treatment strategies for malignant tumors, such as chemotherapy and radiotherapy, have been widely applied in clinical practice. As an excellent drug carrier, COFs can deliver anti-tumor drugs to targets, overcome the limitations of non-specific drug distribution and unfavorable pharmacokinetic properties, and improve therapeutic effects at safe doses. In tumor treatment, COFs can be applied to four common treatment methods as followings.

##### 3.1.1.1 COFs for chemotherapy drug delivery

As a broad-spectrum anti-cancer drug, doxorubicin (DOX) can exert extensive biochemical effects on the body. It shows excellent killing effects on various tumor cells and has a strong cytotoxic effect used in clinical practice. Liu et al. developed COF nanocarriers to effectively encapsulate DOX through hydrophobic and π-π stacking interactions (Liu et al., 2020). After being taken up by tumor cells, this product could exhibite a structural disintegration and release loaded DOX rapidly in tumor cells. Zhang et al. constructed pH-responsive and mitochondrial-targeted methylated COF delivery systems loaded with DOX/camptothecin, achieving a pH-responsive continuous release of DOX/camptothecin and dual-drug delivery (Zhang et al., 2022).

**FIGURE 5:**
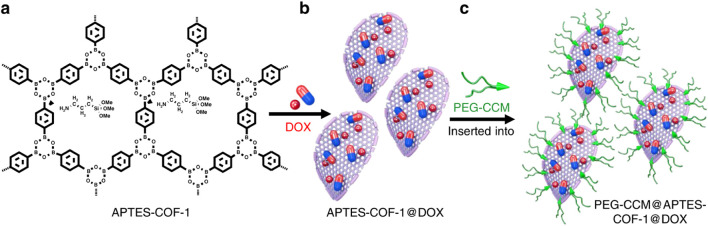
Synthesis of PEG-CCM@APTES-COF-1@DOX for cancer therapy (Li et al., 2017). APTES-COF-1 **(a)** was applied to ecapsulate DOX as APTES-COF-1@DOX **(b)**. APTES-COF-1@DOX and PEG-CCM were self-assembled as PEG-CCM@APTES-COF-1@DOX **(c)**. Reproduced from (Li et al., 2017). Copyright 2017 American Chemical Society.

Zhang et al. developed water-dispersible PEG-CCM@APTES-COF-1 (Figure 5) by self-assembling polyethylene glycol-modified mono-functional curcumin derivatives (PEG-CCM) and amine-functionalized COFs (Zhang et al., 2018). PEG-CCM-modified APTE-COF-1 significantly improved material compatibility, prolonging circulation time in blood. Fluorescence imaging results of drug distribution and *in vitro* distribution showed efficient drug accumulation of PEG2000-CCM@APTES-COF-1@DOX with an excellent cancer suppression.

**FIGURE 6:**
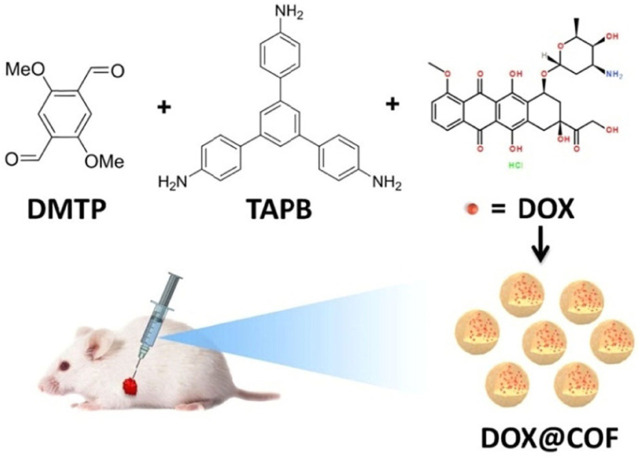
Synthesis diagram of DOX@COF (Liu et al., 2019). DMTP and TAPB were condensed with the encapsulation of DOX. DMTP-TAPB-COF@DOX with 32.1 wt% of drug loading capacity presented superior cancer cell suppression. Reproduced from (Liu et al., 2019). Copyright 2019 Chemistry.

At room temperature, dimethoxy-p-benzaldehyde (DMTP) and tri (4-aminophenyl) benzene (TAPB) were utilized by Liu et al. to synthesize highly monodisperse TAPB-DMTP-COFs through Schiff base condensation (Shi et al., 2017; Xu et al., 2015). TAPB-DMTP-COF@DOX could enhance drug loading capacity with shortened drug sorption time (Figure 6) (Liu et al., 2019). When pH was 5.0 or 6.5, DOX was rapidly released within the first 2 h and the release process was completed in 24 h with a relatively fast release rate. By further lowering the pH value or increasing the reaction time, the complete decomposition of DOX@COF could be achieved in PBS, resulting in the complete release of DOX and demonstrating pH response characteristics. DOX@COF could be used as an efficient chemotherapy platform for kill cancer cells (Gao et al., 2021).

**FIGURE 7:**
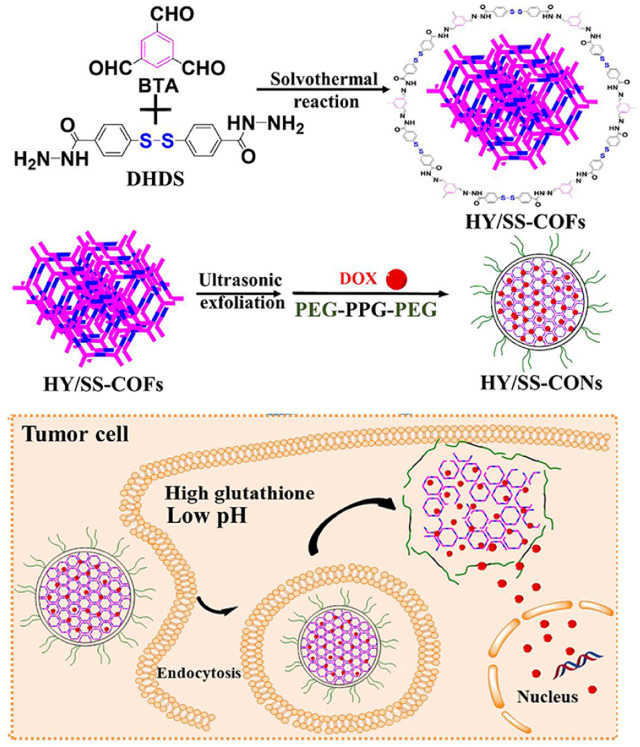
HY/SS-CONs@DOX synthesis and intracellular responsive DOX release (Wang et al., 2020). Schiff reaction of BTA and DHDS achieved the preparation of Poloxamer 188-assembled HY/SS-CONs with encapsulating DOX. HY/SS-CONs can efficiently load DOX due to their porous structure and high surface area, as well as the hydrophobic and π-π stacking interactions between HY/SS-CONs and DOX. Reproduced from (Wang et al., 2020). Copyright 2020 Frontiers in Chemistry.

Wang et al. reported hydrazone and disulfide-containing COFs (HY/SS-COFs) to effectively load and deliver DOX with dual redox and pH sensitivity properties as shown in Figure 7 (Wang et al., 2020). Benzene-1,3,5-tricarbaldehyde (BTA) and 4,4′-Dihydrazide diphenyl disulfide (DHDS) were utilized to prepare HY/SS-COFs. In cancer cell intracellular microenvironment, HY/SS-COFs rapidly disintegrated and effectively released DOX. HY/SS-COFs presented a simple preparation process, controllable morphology and good biocompatibility. Under extracellular conditions, it could achieve a high DOX loading amount with an excellent stability. However, it could achieve the rapid intracellular administration, thereby exerting an effective anti-tumor activity on tumor cells.

Anbazhagan et al. prepared TCOF through the Huisgen cycloaddition reaction between alkynes and azides, modified TCOF with polyethylene glycol, and used the modified TCOF as a carrier for DOX delivery (Anbazhagan et al., 2021). *In vitro* results, DOX could be continuously released from the designed TCOF carrier. DOX sorption on COFs in a water environment was evaluated through molecular dynamic simulation experiments (Ghahari et al., 2021). Drugs were easily adsorbed on the FCOF surface than on the original surface of COFs. Benyettou et al. developed a magnetic nCOFs composite material that could be used simultaneously for MRI, chemotherapy and hyperthermia (Benyettou et al., 2020). This system consisted of porous imine COF particles as drug warehouses and iron oxide nanoparticles as magnetic resonance imaging probes. It was composed of magnetic field-responsive materials and poly (L-lysine) cationic polymers as stabilizers and pH responders. Magnetic nCOFs with magnetism and functional groups were constructed, which would treat diseases such as glioblastoma.

##### 3.1.1.2 COFs for the drug delivery of photodynamic therapy

**FIGURE 8:**
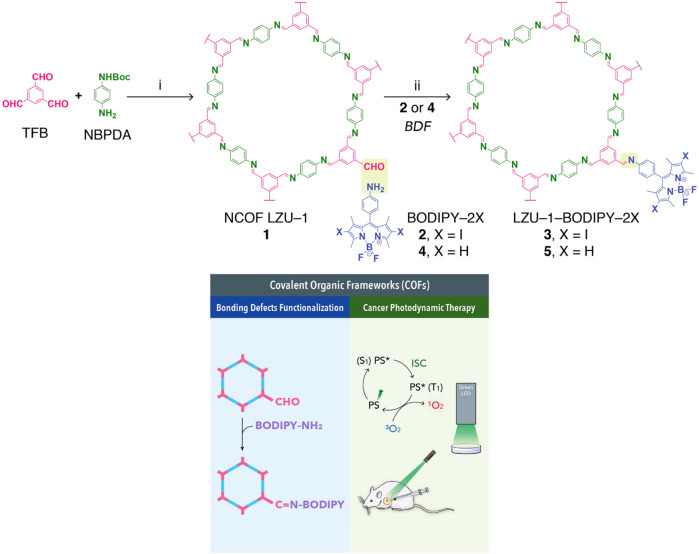
LZU-1-BODIPY-2I and LZU-1-BODIPY-2H for photodynamic therapy (Guan et al., 2019). BODIPY-modified NCOFs were produced from NCOF LZU-1 (1), *BODIPY-2I* (2) and *BODIPY-2H* (4). Markedly, the obtained nanoscale LZU-1-BODIPY-2I (3) and LZU-1-BODIPY-2H (5) are highly crystalline and isostructural to pristine 1, and they both feature low cytotoxicity, good biocompatibility, high cancer cell uptake, and highly efficient ^1^O_2_ generation. Therefore, they can be used as high-performing PDT agents for cancer treatment under the given *in vitro* and *in vivo* conditions. Reproduced from (Guan et al., 2019). Copyright 2019 iScience.

By generating reactive oxygen species (ROS) from photosensitizer via light irradiation at specific wavelengths, photodynamic therapy promotes the cell apoptosis and achieving the goal of killing tumor cells. Given its features such as high surface area and adjustable pore size, COFs have an excellent photosensitizer loading capacity. Moreover, some functional groups existing on COF are conducive to coupling reactions with photosensitizers. Guan et al. obtained two nano-scale spherical COFs functionalized by boron-dipyrromethene (BODIPY) through condensation of imine COFs with bonding defects and amino-substituted boron-dipyrromethene photosensitizers (Guan et al., 2019). Among them, as iodine atom replace one hydrogen atom of BODIPY connected in LZU-1-BODIPY-2H, LZU-1-BODIPY-2I was obtained. This COF drug delivery system with excellent photochemical and dispersion stability showed excellent efficiency to produce ROS (Figure 8). The results of *in vivo* experiments indicated that both these two COFs exhibited relatively high photodynamic therapy efficacies. This was the first time that COFs was reported for the photodynamic therapy of tumors.

##### 3.1.1.3 COFs for the drug delivery of photothermal therapy

Photothermal therapy is a light-based tumor treatment method. Under near-infrared light irradiation, the photothermal agent converts laser energy into heat, thereby raising the temperature of tumor tissues and effectively killing tumor cells. In photothermal therapy, COFs have been used as modifiers to improve the photothermal effect of nanosystems and served as carriers for delivering photothermal agents. Wang et al. synthesized a pH-responsive COF material and encapsulated the photothermal agent heteropoly blue (HPB) in the COF by one-pot method to form HPB@COF nanoparticles *in situ* (Wang et al., 2022). After being taken up by tumor cells, HPB@COF nanoparticles release HPB, which presented a photothermal conversion under 808 nm near-infrared laser, raising the temperature of tumor site and killing tumor cells through high temperature. *In vitro* and *in vivo* test results showed that HPB@COF nanoparticles exhibited an ideal biocompatibility, pH-responsive release and high tumor suppression efficiency, effectively inhibiting the growth of tumors. Fe_3_O_4_@COF prepared by Tan et al. using the template-mediated co-precipitation method could rapidly convert near-infrared energy into local heat. Photothermal conversion efficiency (20.5%) was achieved, demonstrating a potential for the treatment of tumors based on the photothermal therapy regimen (Tan et al., 2016).

##### 3.1.1.4 COFs for the drug delivery of synergistic tumor therapy

Synergistic therapy of tumors is a comprehensive strategy to intervene in multiple characteristics and mechanisms of tumor cells. This therapy can reduce the adverse reactions and effectively inhibit the growth, spread and recurrence of tumors. The modifiability and strong loading capacity of COFs have been widely applied in synergistic therapy of tumors. Hu et al. prepared COFs at room temperature and coupled them with copper selenide (CuSe). These products exhibited high photothermal conversion efficiency and photodynamic therapy effect, successfully achieving the photothermal and photodynamic synergistic therapy (Hu et al., 2019). Wang et al. assembled cyanine dye IR783 with COFs and successfully prepared water-dispersible nanocomposites COF@IR783 (Wang et al., 2019). COF@IR783 presented an excellent photothermal conversion efficiency. Meanwhile, it could be used as a delivery carrier carrying the anti-tumor prodrug cis-aconityl-doxorubicin to achieve the combined treatment of photothermal therapy and chemotherapy. Azo-loaded COFs was prepared as hypoxia-responsive COFs and co-loaded the photodynamic therapeutic agent dihydroporphyrin e6 (chlorine 6, Ce6) and the hypoxia-activated antitumor prodrug tilazamin (Ge et al., 2021). The combination of this photodynamic therapy with the reductase sensitive chemotherapy drug tilazamin could achieve the combined treatment of photodynamic therapy and chemotherapy.

#### 3.1.2 COFs for the delivery of anti-bacteria drugs

The rich pore structure and conjugated structure of COFs make it possible to deliver antibacterial drugs. Their applications often focus on the delivery of effective photosensitizers for photodynamic inactivation of bacteria and antibacterial drugs. Liu et al. prepared two types of COF materials including COFs-Trif-Benz and COF-sDU1 using tris-(4-aldehyde phenoxy)-1,3,5-triazine, benzidine and p-phenylenediamine, respectively through solvothermal reactions (Liu et al., 2017). COFs-Trif-Benz and COF-sDU1 could be used as effective type II photosensitizers for the bacterial photodynamic inactivation. More than 90% of bacteria could be killed. Hynek et al. synthesized a three-dimensional diamond-structured porphyrin-based COF, which could effectively generate O_2_ under the visible light irradiation, especially having a strong destructive effect on the biofilms of *Pseudomonas aeruginosa* and *Escherichia coli* (Hynek et al., 2018). Salehi et al. prepared a highly crystalline COF material using a simple two-step self-assembly method and loaded the antibacterial drug trimethoprim (TMP) to fabricate TMP@COF (Salehi et al., 2022). TMP@COF could efficiently inhibit both gram-negative and gram-positive bacteria and was expected to become a new type of candidate nanomedicine delivery system for the delivery of antibacterial drugs.

#### 3.1.3 COFs for the delivery of anti-inflammatory drugs

Research on the use of COF as a delivery carrier for anti-inflammatory drugs is still in its infancy. Jaggarapu et al. reported that imine-type COFs were synthesized at room temperature and used to load kynurenine (KyH) to obtain COF-KyH (Jaggarapu et al., 2023). COF-KyH could release and regulate the frequency of T cells in rheumatoid arthritis model mice in a controllable manner. Compared with the use of KyH alone, the absorbance value of KyH in the serum of mice orally administered COF-KyH was approximately 10 times higher. Ding et al. prepared porphyrin-COF (DhaTph) membranes by the interfacial polymerization and used them to load IBU to obtain IBU@DhaTph membranes, which were further fabricated into dressings similar to “band-aids” (Ding et al., 2021). The chronic wound healing test on mice fully demonstrated that this product exhibited excellent anti-infection and tissue remodeling activities, which could accelerate the wound healing, promote the formation of new granulation tissue and the growth of new epidermis. Zou et al. prepared curcumin-loaded COFs through condensation reactions. COFs incorporated polycaprolactone nanofiber membranes accelerated the wound healing and skin regeneration in rats, exhibiting great potentials as multifunctional dressings to promote wound healing and skin regeneration (Zou et al., 2022).

**FIGURE 9:**
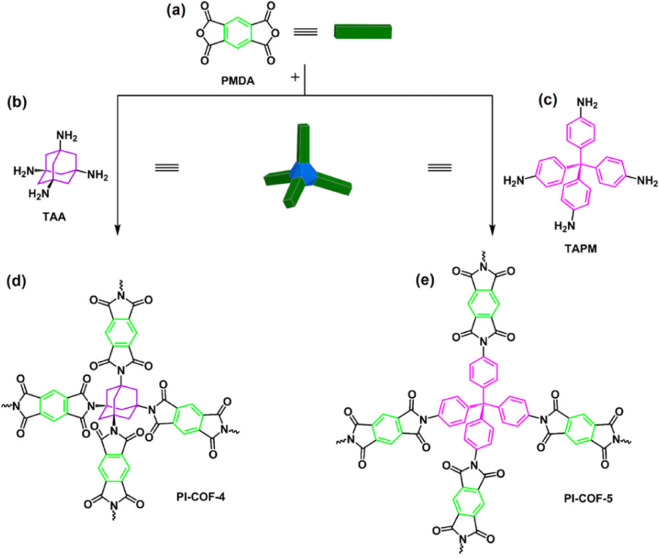
Strategy for preparing 3D porous crystalline polyimide COFs (Fang et al., 2015). TAPM, TAA and PMDA **(a–c)** were utilized to prepare PI-COF-4 **(d)** and PI-COF-5 **(e)**. Reproduced from (Fang et al., 2015). Copyright 2019 American Chemical Society.

Ibuprofen (IBU) belongs to the category of antipyretic and analgesic drugs. Since the biological half-life of IBU is only 2 h, it is often used as a model drug for controlled-release or sustained-release research. In 2015, novel polyimide COFs were prepared by imimination reactions, where PMDA was reacted with tetraminoadamantane (TAA) and tetra-(4-aminobenzene) methane (TAPM) respectively (Figure 9) (Fang et al., 2015). Obtained products including PI-COF-4 and PI-COF-5 with non-permeable and interpermeable structures were shown as uniform rectangular crystals in SEM images and had high thermal stability and surface area. Drug loading amounts of PI-COF-4 and PI-COF-5 were 24 wt% and 20 wt%, respectively. After 12 h, the IBU release rate of PI-COF-4 was 60%, while that of PI-COF-5 was 49%. For the two types of PI-COFs, most of the IBU was released approximately 6 days later, and the total delivery volume could reach about 95% of the initial IBU loading, showing high loading capacity and good controlled-release characteristics.

**FIGURE 10:**
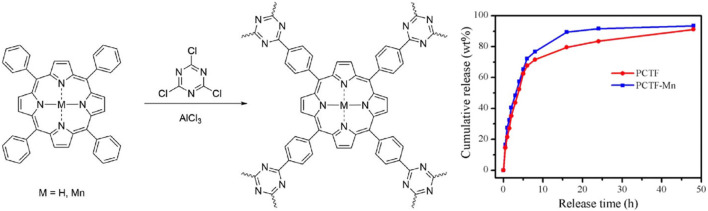
Preparation of two covalent triazine structures based on porphyrin via the Friedel-Crafts reaction (Luo et al., 2017). IBU was loaded with a capacity of 20% and 24% in PCTF and PCTF-Mn via simple immersion. Reproduced from (Luo et al., 2017). Copyright 2017 Journal of Polymer Science Part A: Polymer Chemistry.

Two covalent triazine structures were prepared based on porphyrin, namely, PCTF and PCTF-MN (Figure 10) through the Friedel-Crafts reaction (Luo et al., 2017). This reaction was mild in condition, low in cost and did not require toxic catalysts. Field emission scanning electron microscopy (FESEM) revealed irregular shapes of PCTF. PCTF-MN plates a developed porosity. Release rates of drugs for two PCTFs were almost similar. Release of most loading IBUs finished within 2 days. 89% and 94% of initial IBU loaded amounts could be released.

### 3.2 Applications of MOFs in drug delivery

**FIGURE 11:**
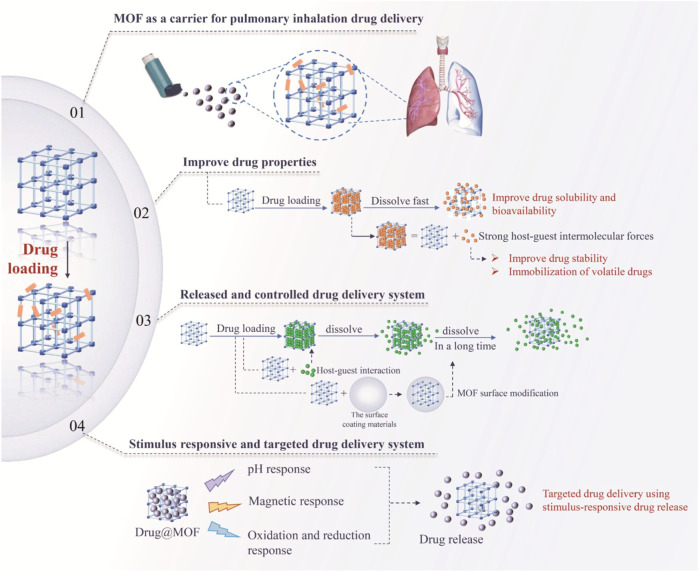
Applications of MOFs in drug delivery system (Tse et al., 2022). The contents included applications of MOFs in pulmonary inhalation drug delivery, improved drug properties of MOFs, construction of released and controlled delivery system, and construction of stimulus responsive and targeted drug delivery system. Reproduced from (Tse et al., 2022). Copyright 2022 American Chemical Society.

The application of MOFs in drug delivery is shown (Figure 11), which was specifically reviewed as following four aspects.

#### 3.2.1 MOFs as carriers for dry powder lung inhalation preparations

MOFs are suitable for the usage as carriers in dry powder lung inhalation preparations. CD-MOF prepared by Tse et al. using the reverse solvent crystallization method has a regular spherical shape, low density, a geometric median particle size (D50) of less than 5 μm, and a fine particle fraction as high as 35% (Zhao et al., 2020). Loading drugs such as ketoprofen, D-limonene, levofloxacin, baicalin, dexamethasone and curcumin on γ-CD-MOF could promote drug absorption in larynx and lungs (Chen et al., 2021; Zhou et al., 2020). The lung toxicity of these drug-loaded MOFs has been proved to be acceptable.

#### 3.2.2 Improvement of pharmaceutical properties by MOFs

MOFs were available to encapsulate drugs with poor solubility in their developed pores. Confined drugs could be released rapidly from MOFs into the dissolution medium. For instance, after loading the insoluble drug isosteviol (STV) into CD-MOF, the solubility of STV in water increased by 1.54 × 10^5^ times compared with the pure drug (Cheng et al., 2020; Lv et al., 2017). At pH 1.0, 4.5 and 6.8, the solubility increased by 1.3 × 10^5^, 1.2 × 10^5^ and 5.49 × 10^5^ times, respectively. The bioavailability of STV in rats increased by 8.67 times than that of original STV.

After loading drugs, MOFs can provide a microenvironment for stable drug storage through the relatively strong intermolecular interaction forces between internal groups and drug molecules. Drug molecules could be bound and the influence of other physical and chemical factors on drugs could be reduced, thereby significantly improving the stability of drugs. Sucralose was prone to degrade under high heat conditions with a degradation rate as high as 86.2% within 1 h at 90 °C (Lin et al., 2016). However, after being loaded into CD-MOF, the thermal stability of sucralose was significantly improved, and the degradation rate within 24 h at 90^o^C was only 13.7%. Moreover, MOFs also have the potential as excipients for drugs to improve the film-forming property of drugs. A new Man@CaCl_2_ MOFs excipient with the typical tablet filler β-mannitol and CaCl_2_ could significantly enhance the mechanical properties of mannitol. Using the drugs with poor compressibility, lurasidone hydrochloride and lornoxicam as models, their application prospects in the tablet pressing process of drug preparations were verified. These works provide valuable references for the inclusion of MOFs in the selection of new pharmaceutical excipients.

#### 3.2.3 MOFs in controlled release drug delivery

As drug carriers, MOFs can avoid the “burst effect” of drugs, control the release of drugs and prolong the drug retention time in various ways. The realization methods mainly include host-guest molecular interactions, surface modification of MOFs, and defect regulation of MOFs.

##### 3.2.3.1 Controlled-release drug delivery via the interaction between host-guest molecules of MOFs-drugs

Drug molecules are often loaded onto the pores or surfaces of MOFs through host-guest intermolecular forces. These would make MOFs available as sustained-release and controlled-release delivery carriers of drugs. For instance, the porphyrin MOFs (i.e., PCN-221) with low cytotoxicity has a high drug loading capacity for methotrexate (Jiang et al., 2022). Methotrexate diffuses into the pores and channels of PCN-221 and achieves sustained-release behavior under physiological conditions through π-π interaction and hydrogen bond interaction with PCN-221.

##### 3.2.3.2 The surface modification of MOFs for controlled-release drug delivery

The physiological environment of human body is complex. It is difficult to achieve the precise and controllable delivery within complex physiological microenvironments merely by controlling host-guest molecule interaction of MOFs/drugs. The surface modification after synthesis of MOFs is a good way to achieve its precise delivery as a controlled release carrier of drugs.

Polyethylene glycol (PEG) surface coating significantly enhanced the chemical stability of procainamide (PA) formulation PA@ZJU-64-NSN in gastric juice, and its drug release process could be triggered by endogenous Na^+^ in target intestinal environments (Li et al., 2020). Pharmacokinetic analysis of chitosan-coated 5-FU@Zr-NDC MOF verified only 20% released 5-FU in an acidic environment, while it reached 70% in artificial intestinal fluid, demonstrating a good controlled-release effect (Teplensky et al., 2017).

##### 3.2.3.3 Defect regulation of MOFs for controlled-release drug delivery

The controlled-release effects of drugs can also be achieved through the defect regulation of MOFs. Teplensky et al. developed a defect regulation scheme that could cause partial pore collapse in NU-1000 and NU-901. Effective convey of model drugs including calcium yellow green pigment and α-cyano-4-hydroxycinnamic acid could be delayed by approximately 2–7 days with complete release only after 30–49 days (Zhang et al., 2017).

#### 3.2.4 Stimulus-responsive MOF carriers for targeted drug delivery

Stimulus-responsive carriers refer to a type of carrier that can generate rapid and precise responses to external stimuli such as temperature, magnetic fields, ultrasound, light, pH, etc., thereby controlling the presentation and release of drugs. When MOFs are used as drug carriers, it is usually hoped that they have both targeting and stimulus-responsive properties to improve their bioavailability.

##### 3.2.4.1 pH-responsive MOFs for targeted drug release

MOFs can be used to dissolve the framework structure and release drugs in special pH microenvironments around the target cells. For instance, ZIF-90 could maintain its structural stability in neutral and slightly alkaline environments. Therefore, the drug release of ZIF-90 loaded with doxorubicin and 5-FU in the tumor microenvironment (pH 5.5) could reach more than 95% (Leng et al., 2018). MIL-101(Fe) exhibited open metallic sites. Curcumin was successfully encapsulated in MIL-101(Fe) by adsorbing onto this metallic site. MIL-101(Fe) presented a higher release rate under acidic conditions and was released slowly in a normal cellular environment (Yang et al., 2017).

##### 3.2.4.2 Magnetic response of MOFs for targeted drug release

After entering the body, magnetic response nanocarriers can achieve the magnetic-mediated drug therapy through the action of exogenous magnetic fields. If magnetic response nanocarriers mediate the treatment of tumors through the magnetic resonance imaging, it can integrate the diagnosis and treatment into a single system. For instance, Fe-MOF modified with pH-sensitive hydroxyapatite (HAp) and magnetic core (Fe_3_O_4_) presented excellent biocompatibility and tumor targeting properties when it was used for the magnetic targeted drug delivery and pH-controlled drug release (Curcio et al., 2017).

##### 3.2.4.3 Redox response of MOFs for targeted drug release

The concentration of reducing agent glutathione (GSH) within tumor cells was more than 1 × 10^3^ times that outside the cells and 4 times higher compared with inside ones. Concentrated GSH could trigger the breaking of disulfide bonds in drugs, thereby causing their release in the cytoplasm (Wu et al., 2022). This provided an idea for designing reduction-responsive drug carriers. Imidazole containing thiol bonds were combined with Mn to load cisplatin (CDDP), and the nanoengineering was carried out on phase change materials (PCM) with homologous targeting and tumor cell membranes (TCM). Mn-MOF-CDDP@PCM-TCM nanomissile was ultimately prepared. Mn-MOF-CDDP@PCM-TCM could achieve microwave thermodynamic chemotherapy (Cai et al., 2021).

##### 3.2.4.4 Other stimulus responses of MOFs for targeted drug delivery

Active targeting refers to an autonomous behavior in which nanoparticles rely on the recognition ability of targeting molecules to enter, bind and act on specific targets. It is usually achieved through methods such as surface functionalization of nanomaterials and targeted molecular grafting. MIL-101(Fe) modified with folic acid and 5-carboxyluciferin (5-FAM) could be used for the targeted delivery of anti-tumor drug triptolide. Compared with MIL-101(Fe), functionalized MIL-101(Fe) nanoparticles not only exhibited better targeting efficiency but also reduced the systemic toxicity of the drug (Xue et al., 2019). The surface modification of MIL-100 loaded with doxorubicin (DOX) by hyaluronic acid (HA) released the drug in a pH-dependent manner due to the characteristics of MIL-100, and exhibited the ability to target tumor tissues due to the HA modification on the surface. Folic acid-anchored MOFs were applied for tumor-targeted drug delivery (Alves et al., 2021). By adopting a reasonable strategy, the two-photon-active PCN-58-Ps was prepared through click chemistry, and the specific targeting characteristics of cancer cells were obtained by covering it with HA through the coordination (Li et al., 2021).

### 3.3 Therapeutic pharmaceutical property comparison

Comparison of therapeutic effects and pharmaceutical properties of MOFs and COFs is presented as following.

For therapeutic effect, MOFs present diverse functions including serving as a carrier and providing metal nodes with have therapeutic activities such as antibacterial and anti-tumor properties. For instance, MOFs can kill bacteria by controlling the release of metal ions or generating reactive oxygen species. However, the presence of metal ions from MOFs may raise concerns about biological safety. The long-term effects and potential toxicity require more rigorous assessment. COFs achieve the therapeutic effect by loading chemotherapeutic drugs, photosensitizers, etc. In photodynamic therapy and photothermal therapy, the structure of COFs is conducive to energy transfer, demonstrating great potential. Whereas, COFs lack the inherent therapeutic activity like MOFs, and its functions are entirely dependent on “loading” and “modification”.

For drug-loading capacity, MOFs present high drug loading capacity due to abundant pore structures. The pore size and surface properties can be highly adjusted, making it suitable for loading various drugs ranging from small molecules to proteins. There is no obvious disadvantage for drug-loading capacity of MOFs. COFs with high specific surface area present extremely high drug loading capacity. The precise pores and adjustable framework chemical properties of COFs enable the drug loading capacity for chemotherapy drugs to reach up to 70–90 wt%. However, the pore size or stability of some early COFs (such as borate esters) may not be ideal.

For drug release, the response of MOFs to stimulation is strong. The coordination bond characteristics of MOFs make it sensitive to various pathological microenvironmental changes (e.g., pH value, ions, and oxidation-reduction). MOFs can achieve “on-demand” precise release. However, MOFs may release prematurely due to insufficient stability, and the release may not be complete. COFs can also achieve stimulus-responsive release (e.g., pH and light response). By reasonably designing the covalent bond connection, the controlled and sustained release of drugs by COFs can be achieved. Whereas, the response speed and sensitivity of COFs may be inferior to those of MOFs based on coordination bonds.

For biological safety, some MOFs (e.g., ZIF-8, UiO-66) exhibit controllable degradation behavior under physiological conditions, demonstrating a good short-term biocompatibility. However, the core challenge lies in the potential long-term toxicity of metal ions from MOFs and the unclear metabolism in the body, which is one of the main obstacles for clinical applications. COFs contain no metals at all and are composed of common light elements found in living organisms. It typically exhibits higher biocompatibility and lower immunogenicity. Whereas, COFs lack natural pathways for in-body degradation and clearance, and the long-term fate and metabolic process in the body are still unclear.

For stability, many MOFs (e.g., Zr-MOFs) have good stability in physiological conditions, which can protect the drugs from being damaged during the circulation process. However, some MOFs have poor stability in water or acidic environments and are prone to structural collapse. COFs connected by strong covalent bonds present high chemical stability, and be able to maintain the structural integrity in various environments (including water, acids, and bases). Whereas, excessive stability of COFs may make it difficult to be degraded and metabolized in the body. Early borate ester COFs were sensitive to water, and have gradually been replaced by more stable imine types, etc.

## 4 Conclusions and prospects

COFs and MOFs with high stability, developed porosity, light weight and diverse structure present great prospects for drug delivery. This review analyzes the internal reasons of COFs and MOFs as drug delivery carriers from two perspectives: the challenges they face in research and the relevant policies and regulations for drug marketing, providing ideas for promoting their application in actual drug development.

Development times of COFs and MOFs are short. Compared with the industrial application of other traditional porous materials, they are still far behind and still face a large number of challenges in many aspects. For instance, how to design and synthesize COFs and MOFs with excellent performance, how to improve comprehensive performances and industrial practicality, etc. Due to the fact that the performances of COFs and MOFs in practical applications are not perfect, this points out the main research directions for us in the future. Potential directions including green synthesis methods and developing strategies to overcome immunogenicity challenges *in vivo* were proposed as following.

1. Exploring green synthesis methods for MOFs and COFs is crucial for promoting these two types of highly promising porous materials from the laboratory to large-scale applications. Taditional solvothermal method induced heavy environmental burdens. Core objective is to avoid toxic solutions, decrease energy consumption, and utilize renewable raw materials to promote these high-performance materials from the laboratory to sustainable industrial applications. Recently, several green approaches have been developed including Vapor-phase assisted method, solvent-free/mechanical chemistry method, and micro-plasma electrochemical method. For vapor-phase assisted method, the solid metal source and organic ligands are placed in a container with only a small amount of solvent added (or the reaction atmosphere is provided solely by its vapor). This approach can significantly reduce the solvent amount, and be applicable for the preparation of high-quality MOF films and composites with simplified process. For solvent-free/mechanical chemistry method, the reaction is driven by mechanical grinding or melting without using any solvents. For micro-plasma electrochemical method, a micro-plasma electrochemical strategy has been developed for rapidly synthesizing COFs under normal temperature and pressure. This method can complete the reaction in just a few minutes. Its space-time yield is 1,000 times higher than that of traditional solvent heating methods, and can be carried out in an aqueous medium, completely avoiding harmful organic solvents.

Exploring the green synthesis methods will continue to evolve towards greater efficiency, greater universality, and greater scalability. They will also be combined with tools such as life cycle assessment (LCA) to comprehensively evaluate their environmental benefits, ultimately promoting the sustainable application of MOFs/COFs.

2. Overcoming the immunogenicity challenges of MOFs and COFs *in vivo* is a current cutting-edge topic in the field of biomedicine. The challenge here is actually twofold. Unnecessary immune attacks of MOFs and COFs should be avoided. On the other hand, it is necessary to precisely control the antigens or drugs delivered by MOFs and COFs to induce the desired immune response. Several strategies have been developed including precisely regulating the type of immune response, camouflage and shielding via bionic functionalization, and source design and surface treatment. For precisely regulating the type of immune response, the release rate of antigens can be precisely controlled by altering the pore size of MOFs/COFs, thereby “training” the immune system to generate specific types of T cell responses. For camouflage and shielding via bionic functionalization, MOFs/COFs could be wrapped with cell membranes of immune cells or tumor cells to enhance the stability of MOFs/COFs and reduce the immunogenicity. For source design and surface treatment, MOFs could be constructed using low-toxicity metals such as iron and gallium, and COFs could be composed entirely of light elements to prevent the release of toxic metal ions at the source, thereby avoiding the immunological risks associated with such releases. Moreover, hydrophilic polymers could be connected to the surface of MOFs/COFs to form a “hydration layer”, which shields the surface charges and reduces non-specific protein adsorption, thus preventing MOFs/COFs from being engulfed by immune cells as a foreign substance.

Taken together, future research will focus on designing an integrated intelligent nanosystem with a biomimetic coating to respond to the tumor microenvironment and induce immunogenic cell death. The success of these strategies would significantly drive the transformation of MOFs/COFs towards cancer immunotherapy, novel vaccine adjuvants, and other directions.
